# Sulfhydrated albumin transmits H_2_S signaling and ameliorates DOX-induced multiorgan injuries

**DOI:** 10.1016/j.redox.2025.103631

**Published:** 2025-04-08

**Authors:** Yijun Xu, Yang Sui, Rui Jiang, Xin Wang, Mika Suda, Manabu Niimi, Zhimin Mao, Zhen Zhang, Shao-Ling Zhang, Jianglin Fan, Jian Yao

**Affiliations:** a Division of Molecular Signaling, Department of the Advanced Biomedical Research, Interdisciplinary Graduate School of Medicine, University of Yamanashi, Chuo, 409-3898, Japan; b Division of Molecular Pathology, Interdisciplinary Graduate School of Medicine, University of Yamanashi, Chuo, 409-3898, Japan; c Department of Medicine, Université de Montréal, Centre de recherche du Centre hospitalier de l'Université de Montréal (CRCHUM), 900 Saint Denis Street, H2 X 0A9, Montréal, QC, Canada; d Guangdong Province Key Laboratory, Southern China Institute of Large Animal Models for Biomedicine, School of Pharmacy and Food Engineering, Wuyi University, Jiangmen, China

**Keywords:** Protein sulfhydration, Sulfhydrated albumin, H_2_S, cAMP signaling pathway, Inflammatory responses, Doxorubicin toxicity

## Abstract

Hydrogen sulfide (H_2_S) is a vital signaling molecule involved in various physiological processes; however, the mechanisms underlying its systemic signaling remain poorly understood. We hypothesized that albumin, the predominant plasma protein and a vital sulfhydryl carrier, mediated systemic H_2_S signaling, which could potentially treat H_2_S-deficient diseases. This study aimed to investigate this hypothesis. Our results showed the presence of sulfhydrated proteins in normal mouse serum, with albumin being particularly enriched. The level of sulfhydration was influenced by H_2_S availability and the redox environment. In vitro incubation of albumin with NaHS resulted in an increased number of sulfhydrated groups. Under reductive conditions, this sulfhydrated albumin (–SSH–Alb) released substantial amounts of H_2_S. When –SSH–Alb was added to cultured endothelial cells, it activated the cAMP signaling pathway, upregulated cystathionine γ-lyase (CSE) expression, and enhanced intracellular H_2_S levels. In an in vitro inflammatory model involving macrophages and endothelial cells, –SSH–Alb inhibited macrophage adhesion, reduced LPS-induced expression of adhesion molecules, and suppressed cytokine production and inflammasome activation. These effects correlated with improved cellular redox status. Furthermore, in vivo administration of –SSH–Alb protected mice from doxorubicin (DOX)-induced cardiotoxicity and intestinal damage. It improved mouse mortality, and alleviated ferroptotic cardiac injury and gut barrier dysfunction. These therapeutic benefits were associated with rebalanced local and systemic redox status. In summary, our study reveals that –SSH–Alb reserves, transmits, and amplifies H_2_S signals and exhibits significant anti-inflammatory and antioxidant properties. This characteristic of –SSH–Alb holds promise for preventing and treating a wide range of diseases.

## Highlights

• Albumin is a major carrier of protein sulfhydration in blood serum.
• Sulfhydrated albumin (–SSH–Alb) reserves, transmits and amplifies H_2_S signaling.
• -SSH-Alb activates cAMP pathway and stimulates CSE expression.
• -SSH-Alb exhibits potent anti-inflammatory and antioxidant effects.
• -SSH-Alb protects against DOX-induced cardiotoxicity and intestinal injury in mice.

## Graphical abstract

**Figure:**
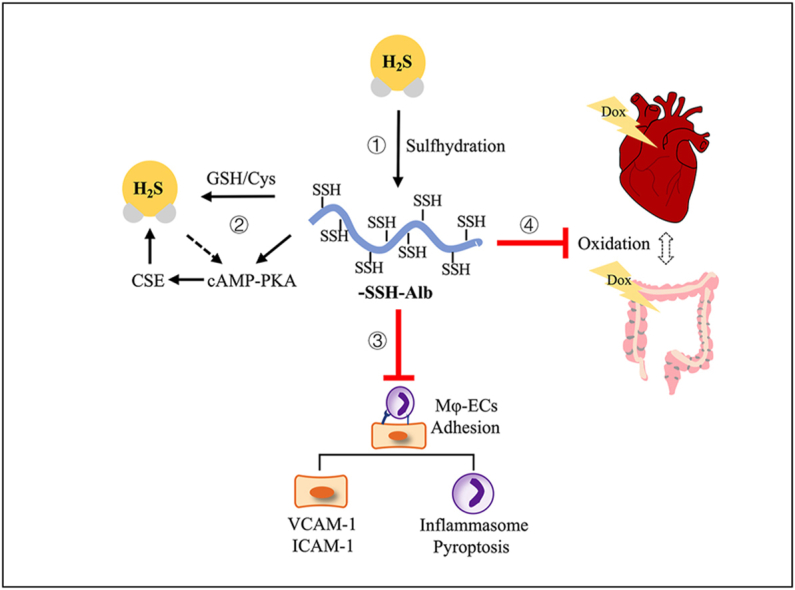

## 1 Introduction

Hydrogen sulfide (H_2_S) is an important gaseous mediator with significant biological activities. In mammals, H_2_S is primarily synthesized by three enzymes: cystathionine β-synthase (CBS), cystathionine γ-lyase (CSE), and 3-mercaptopyruvate transferase (3-MST). H_2_S has diverse physiological functions, including vascular dilation, cardiac protection, neurotransmission, angiogenesis induction, anti-inflammatory and antioxidant effects. A deficiency in H_2_S has been implicated in the onset and progression of various diseases [1].

The biological actions of H_2_S are thought to be mediated mainly through protein sulfhydration [[2], [3], [4]], a post-translational modification of cysteine residues that forms sulfhydrated (-SSH) groups. This modification alters protein structure and function, influencing cellular signaling and phenotype. For instance, H_2_S-induced sulfhydration of human antigen R (HuR) has been shown to mediate its protective effects in colitis [5], while sulfhydration of sirtuin-1 contributes to its anti-atherosclerotic properties [6]. However, our understanding of extracellular protein sulfhydration remains limited despite extensive documentation of intracellular protein sulfhydration. Given that H_2_S has a short half-life and limited diffusion ability and that it has critical biological functions in circulation, such as preventing platelet aggregation [7], the question occurs as how H_2_S, either endogenously produced or exogenously administered, exerts systemic effects. Specifically, how is H_2_S signaling transmitted extracellularly? We speculated that sulfhydrated extracellular proteins, particularly albumin, may be key to this process.

Albumin is the predominant plasma protein in the blood, playing critical roles in maintaining colloid osmotic pressure, binding and transporting various molecules, regulating acid-base balance, and modulating immune responses [8,9]. Notably, albumin contains a single unpaired free cysteine residue (Cys34), which accounts for approximately 90 % of free thiols in the extracellular space [10]. This unique feature endows albumin with potent antioxidant properties and makes it highly susceptible to post-translational oxidative modifications. Elevated levels of oxidized albumin have been linked to the initiation and progression of various diseases [8,11].

Albumin is susceptible to H_2_S-mediated post-translational modification, resulting in the formation of –SSH–Alb [4]. This susceptibility can be attributed to the fact that albumin is predominantly synthesized in the liver, the primary organ responsible for generating the majority of H_2_S in vivo [1]. This co-localization of albumin and H_2_S suggests a high probability of albumin sulfhydration. In line with this view, incubating albumin with the H_2_S donors led to the formation of –SSH–Alb [[12], [13], [14]]. Furthermore, oxidized cysteine residues in Alb can react with H_2_S to form sulfide products (RSSH) [15,16]. Indeed, the presence of sulfhydrated and polysulfidated proteins in serum and tissues, as well as the dynamic changes in sulfhydration levels under pathological conditions [12,17,18], have been reported. Additionally, polysulfidated Alb created through a reaction with sodium polysulfides has been shown to possess anti-oxidative capacity in culture [13]. Beyond H_2_S-related modification, recent studies have identified cysteinyl-tRNA synthetase (CARS2) as a critical enzyme capable of directly modifying proteins during post-translational processes, contributing to protein sulfhydration [19,20]. Considering this background, we hypothesize that sulfhydrated albumin (–SSH–Alb) may serve as a primary carrier of H_2_S in circulation, effectively transmitting systemic H_2_S signals and facilitating essential biological functions. The aim of this study was to investigate this hypothesis and explore the potential application of this property in disease treatment.

Here, we present evidence for the existence of –SSH–Alb in serum, which functioned as a reservoir, mediator, and amplifier of H_2_S signals in vivo. Additionally, it exhibited anti-inflammatory and antioxidative properties, possessing the potential for treating doxorubicin (DOX)-induced cardio and intestinal toxicity. Our findings provided novel mechanistic insights into H_2_S signaling and proposed that –SSH–Alb could be a valuable therapeutic target for H_2_S deficiency-related diseases.

## 2 Material and methods

### 2.1 Materials

Bovine serum albumin (BSA, Fraction V, cat no.A001) was purchased from Iwai Chemical. Company (Tokyo, Japan). Dimedone (cat no.D0701) and doxorubicin (DOX, cat no.D4193) were obtained from Tokyo Chemical Industry (Tokyo, Japan), while Alexa 680 Fluor C2 maleimide (cat no.A20344) was from Thermo Scientific (Rockford, IL, USA). DCP-Rho1(cat no. 13194) was obtained from Cayman Chemical (Ann Arbor, MI, USA). The anti-cysteine sulfenic acid antibody (cat no.07-2139-I) was obtained from Millipore (Burlington, MA, USA). HRP-conjugated anti-rabbit or mouse IgG (cat no.7776S or 7074S), anti-β-actin (cat no.A5316), anti-GPX4 (cat no.52455), anti-GSDMD-NT(cat no.10137), anti-phospho-Ser157 VASP (cat no.3111), and anti-p-P38 antibodies (cat no.9662) were purchased from Cell Signaling, Inc. (Beverly, MA, USA). Anti-NLRP3 (cat no. NBP2-12446) and anti-x-CT (cat no. NB300-317) antibodies were bought from Novus Biologicals (Littleton, CO, USA). The anti-phospho-Ser133 CREB antibody (cat no. 11052) was sourced from Signalway Antibody (College Park, MD, USA). Anti-ICAM-1 and anti-VCAM-1 antibodies (cat no.15364-1-AP and 11444-1-AP) were from Proteintech Group, Inc. (Chicago, IL, USA). Hsip-1 DA (cat no.SB22) was obtained from Dojindo Laboratories (Kumamoto, Japan). Anti-CSE antibody (cat no. sc-374249) was from Santa Cruz Biotechnology, Inc. (Santa Cruz, CA, USA), and the anti-lipocalin-2 antibody (cat no. AG-25A-008) was acquired from Adipogen (San Diego, CA, USA). Anti-E-cadherin antibody (cat no.sc-7870) was sourced from BD Biosciences (NJ, USA). Calcein-AM (cat no.1646257) was purchased from Dojindo Molecular Technologies (MD, USA). Minute™ albumin depletion reagent (cat no.WA-013) was from Invent Biotechnologies, Inc. (Fernbrook Ln N, Plymouth, MN, USA). Sodium hydrosulfide hydrate (NaHS, cat no.161527), glutathione (GSH, cat no.G4251), anti-Cx43 (cat no.C6209), and all other chemicals were obtained from Sigma (Tokyo, Japan).

### 2.2 Animal experiments

Adult male C57BL/6 mice (20–30 g) were housed in a temperature-controlled facility with a 12-h light cycle and provided ad libitum access to standard rodent chow. The animal study was approved by the Animal Care and Use Committee of Yamanashi University and conducted according to established animal experimentation guidelines.

For experiments with DOX cardiotoxicity, mice were randomly divided into three groups (n = 6 per group): negative control, DOX-positive control, and –SSH–Alb-treated DOX group. For induction of acute cardiotoxicity, mice were administrated with a single dose of DOX (25 mg/kg) or an equal volume of normal saline (NS) intraperitoneally (IP). The modified –SSH–Alb was administrated at the dose of 1.5 g/kg for 5 times before and after DOX injection at the interval of 12∼24 h. Control mice received an equivalent volume of NS. All mice were weighed daily and sacrificed on day 3 post-DOX injection. At the endpoint, mice were anesthetized using intraperitoneal sodium pentobarbital (40 mg/kg) (Somnopentyl®, Kyoritsu Seiyaku Corp., Tokyo, Japan), and blood, heart, and colon samples were collected for macroscopic scoring and assessments of oxidative stress and tissue damage.

For studies with TNBS colitis and renal ischemia-reperfusion injury, mice models were established, as we recently reported [21,22]. Briefly, mice were intrarectally administered with 200 mg/kg TNBS (Sigma-Aldrich) dissolved in 50 % ethanol to induce TNBS colitis. For induction of renal ischemia-reperfusion injury, the renal pedicle (artery, vein, and ureter) of both kidneys was clamped for 40 min with clips, followed by removing the clips to recover the normal blood flow. Serum was collected 24 h after the injection or operation.

### 2.3 Human umbilical vein endothelial cell culture

Human umbilical vein endothelial cells (HUVECs) were obtained from PromoCell. (Heidelberg, Germany) and cultured in endothelial cell growth medium 2 (ready-to-use; Takara-Bio Inc), supplemented with 5 % FBS and 1 % antibiotic and antimycotic solution. Cells were seeded into culture plates and allowed to grow to confluence before stimulation with various reagents for the indicated time intervals.

### 2.4 Primary culture of bone marrow-derived macrophages (BMDMs)

Bone marrow-derived macrophages (BMDMs) were obtained from the femurs of mice as previously reported [23]. The femurs were isolated and soaked in Dulbecco's Modified Eagle Medium (DMEM)/F-12 containing 1 % ABAM. The epiphyses were cut off, and bone marrow cells were flushed out using a syringe with a 21G needle. The harvested cells were seeded into a 12-well plate at a density of 1.5–2 × 10**^6^** cells per well and cultured with 20 % FBS and 20 ng/ml M-CSF to induce BMDMs. After three days, the culture medium was exchanged. After four days of culture, non-adherent cells were removed, and adherent macrophages were used for experiments.

### 2.5 Western blot analysis

Western blot analysis was conducted as previously described [24]. Cellular lysates were prepared using 1 × SDS lysis buffer (62.5 mM Tris-HCl, 2 % SDS, 10 % glycerol), while tissue samples were homogenized in RIPA lysis buffer with a freshly added proteinase inhibitor cocktail. Protein concentrations were measured using the Micro BCA Protein Assay Kit (Thermo Fisher Scientific, Waltham, MA). Equal amounts of protein were separated by 10 % SDS-PAGE and transferred to PVDF membranes using a wet-blotting apparatus. After blocking with 5 % skimmed milk or 3 % BSA in 0.1 % Tween-20 PBS solution (PBST) for at least 1 h, membranes were incubated overnight with primary antibodies at the appropriate dilution. Following PBST washes, membranes were incubated with peroxidase-conjugated secondary antibodies for 1 h and detected using an enhanced chemiluminescence system (Nacalai Tesque, Kyoto, Japan). Chemiluminescent signals were captured with a Fujifilm image LAS-1000 analyzer (Fujifilm, Tokyo, Japan), and band intensities were quantified using NIH Image J software (http://rsb.info.nih.gov/ij). EZ blue staining of the membrane protein or probing with β-actin or GAPDH was performed to confirm equal loading of samples.

### 2.6 Preparation of sulfhydrated albumin (–SSH–Alb) and reductively albumin (–SH–Alb)

Bovine serum albumin (BSA), dissolved in distilled water (DW) at 250 mg/ml, was allowed to react with 50 mM DTT at 4 °C overnight to release sulfhydryl groups (-SH). The solution was then put into a dialysis tube (7000 MWCO; Thermo Scientific, Waltham, MA) and dialyzed against a 50∼100-fold volume of distilled water with more six exchanges of dialysis water to completely remove the unreactive DTT and its metabolites. A portion of the –SH–exposed Alb (–SH–Alb) was then treated with 1 mM H_2_O_2_ at room temperature for 1 h to induce thiol oxidation, followed by dialysis and reaction with 50 mM NaHS at 4 °C overnight to induce albumin sulfhydration. After thorough dialysis as described above with a span more than 72 h, the modified proteins were measured for protein concentration, verified for the increased level of –SH and –SSH groups, aliquoted, and stored at −80 °C until its use. The final filtrate was also collected, aliquoted and used to confirm the absence of the bioactive NaHS.

### 2.7 Detection of free sulfhydryl (-SH) groups with maleimide-labeling assay

The protein –SH group was assessed as previously described [21,24]. Protein samples were reacted with Alexa Fluor 680C2 maleimide (red fluorescence, final concentration of 5 μM) at 4 °C for 2 h. After the reaction, protein samples were subjected to SDS-PAGE separation. The signal from the gel was captured using a Fujifilm image LAS-1000 analyzer (Fujifilm, Tokyo, Japan) and quantified with ImageJ software. EZ blue staining was performed to confirm equal loading of proteins.

### 2.8 Dimedone switch method for detection of sulfhydration

The Dimedone switch method was used to detect protein sulfhydration levels as reported by Zivanovic et al. [25] with minor modification. Briefly, cell or tissue lysates, or pure albumin, were incubated with 5 mM NBD-CI in PBS at 37 °C for 30 min. The proteins were then precipitated using a methanol/chloroform precipitation method (Sample/Methanol/Chloroform, 4/4/1, v/v/v) and centrifuged at 13,200 rpm for 20 min at 4 °C. The supernatant was discarded, and the precipitation step was repeated twice. The pellet was washed with methanol 2–3 times, resuspended in 50 mM Hepes containing 2 % SDS, and sonicated for 15 s. The sample was then incubated with 5 μM DCP-Rho1 for 20 min at 37 °C, followed by a mixture with fivefold non-reducing sample buffer and SDS-PAGE separation. The signal of the labeled DCP on the gels was directly captured with a Fujifilm image LAS-1000 analyzer and quantified with ImageJ software. EZ blue staining was performed to confirm equal loading of proteins.

### 2.9 Detection of sulfenic acid (-SOH)

The formation of –SOH in proteins was determined as previously described [21,24]. Serum samples were diluted 20 to 50-folds, and cell and tissue lysates were prepared at 1–5 mg/ml concentrations. Proteins (10–30 μg) were incubated with 1 mM dimedone or 5 μM DCP-Rho1 at room temperature for 20 min to label –SOH. Labeled samples were then mixed with non-reducing SDS sample buffer and subjected to SDS-PAGE followed by Western blotting using an anti-dimedone antibody to detect protein-bound dimedone or direct capture of fluorescent signal of DCP-Rho1 with a Fujifilm image LAS-1000 analyzer.

### 2.10 Induction of protein sulfhydration by in vivo administration of H_2_S

A single intraperitoneal injection of 15 mg/kg of the H_2_S donor NaHS or an equal volume of normal saline to mice was performed. After 3 h, mice were anesthetized, and blood and liver samples were collected and assayed for the sulfhydration levels with the dimedone switch method.

### 2.11 Induction of protein sulfhydration by H_2_S in vitro

Normal mouse serum and liver tissue lysate were incubated with 100 μM NaHS at room temperature for 1 h, followed by centrifugation in a 0.5 ml centrifugal filter tube (Merck KGaA, Darmstadt, Germany) at 13,200 rpm for 20 min. This centrifugation step was repeated six times to remove excess NaHS. The collected serum and liver samples were subsequently tested for sulfhydration.

### 2.12 BMDMs and HUVECs adhesion assay

Endothelial cells, seeded in 96-well plates at a density of 25,000 cells per well, were pretreated with 1.0 mg/ml of differently modified albumins for 30 min, followed by stimulation with LPS at 500 ng/ml for 6 h. Afterward, Calcein-AM-labeled BMDMs were added at a density of 10,000 cells per well and allowed to adhere to endothelial cells for 30 min. After washing out the non-adherent BMDMs, cells attached to endothelial cells were photographed with a fluorescence microscope, and the number was counted.

For preparation of Calcein-AM-labeled BMDM_S_, cells were labeled with 2 μM Calcein-AM for 30 min. After washing away the unlabeled Calcein-AM with PBS, the cells were resuspended in a serum-free medium and used for the above adhesion assay.

### 2.13 Determination of H_2_S production capacity with lead sulfide method

H_2_S production capacity was assayed as we have previously described [26]. Briefly, H_2_S test paper was prepared by soaking the 3 M filter paper in a 20 mM lead acetate solution and dried. To measure H_2_S generating capacity, differently modified albumin was either added into the solution containing 5–10 mM reducing chemicals or cultured HUVECs at the density of 4 × 10^5^ in 96-well culture plates. H_2_S test paper was placed directly onto the 96-well plates for 2–24 h. The reaction of H_2_S with lead acetate in the test paper resulted in the formation of lead sulfide, which left a visible black-colored circle on the paper. The intensity of the color correlates with the produced H_2_S concentration.

### 2.14 Detection of intracellular H_2_S with Hsip-1 DA probe

Hsip-1 DA probe was used to detect intracellular H_2_S level, as we have previously reported [27]. Briefly, HUVECs, pretreated with or without modified albumin, were exposed to 5 μM Hsip-1 DA for 30 min. After washing, fluorescent images of the cells were captured with a fluorescence microscope (IX71, Olympus, Tokyo, Japan), and the cellular intensity of fluorescence was quantified using NIH ImageJ software.

### 2.15 Enzyme-linked immunosorbent assay (ELISA)

The collected cell supernatant was assayed for IL-1β using an ELISA Kit from Peprotech following the manufacturer's instructions (Rocky Hill, NJ, USA) [23,24].

### 2.16 Serum albumin depletion experiment

Serum albumin was depleted using a commercially available albumin depletion reagent (Cat. No. WA-013, MINUTE Albumin Depletion Reagent for Plasma and Serum, Invent Biotechnologies, Inc., Plymouth, MN) according to the manufacturer's instructions [28,29]. Briefly, 50 μl of mouse serum was thoroughly mixed with an equal volume of the albumin depletion reagent. The mixture was centrifuged at 13,200 rpm for 5 min, and the supernatant containing albumin was discarded. The remaining pellet was resuspended and used for subsequent experiments.

### 2.17 Statistical analysis

Values are expressed as mean ± SE. Comparisons between two groups were made using Student's t-test. For multiple comparisons, one-way analysis of variance (ANOVA) was employed. All analyses were performed using Microsoft Excel or GraphPad Prism version 8.0, with statistically significant differences at p < 0.05.

## 3 Results

### 3.1 Protein sulfhydration in serum and its regulation by exogenous H_2_S and under pathological conditions

**Fig. 1:**
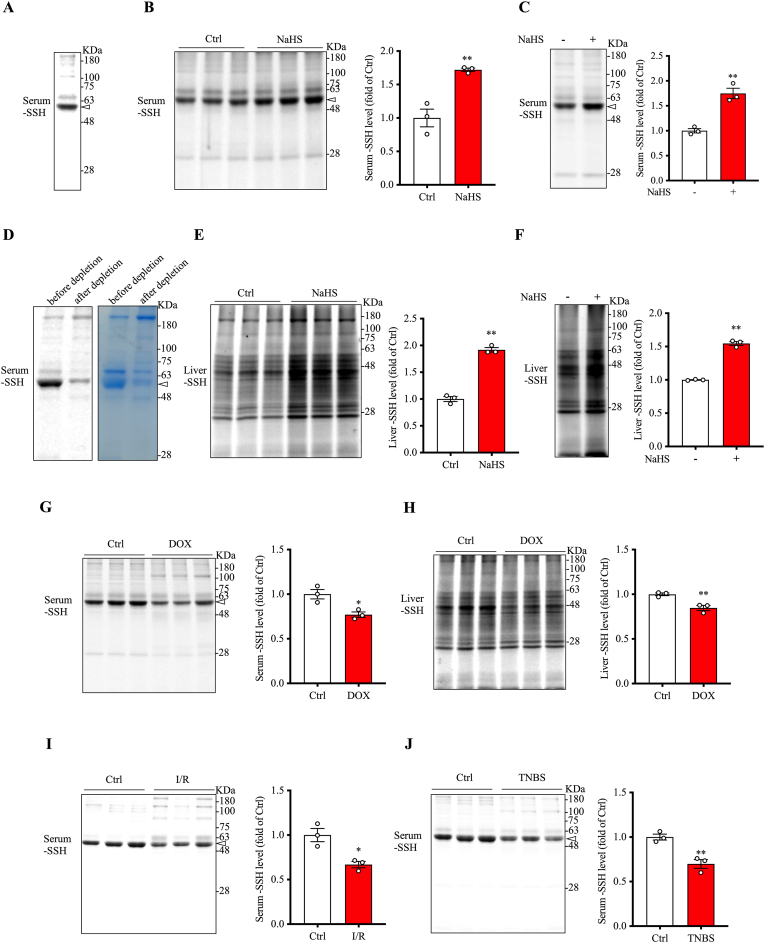
**Protein sulfhydration in serum and liver and its modulation by exogenous H_2_S and under pathological conditions.** (A) Detection of sulfhydrated proteins in serum from normal mice using the dimedone switch method. (B, C) Effect of the H_2_S donor NaHS on serum protein sulfhydration. Sulfhydration was assessed in serum from mice administered with 15 mg/kg NaHS for 3 h (B) or after in vitro incubation with 100 μM NaHS for 1 h (C). Quantification of serum protein sulfhydration at the MW around 65 KDa in the blot of B and C is shown at the right side (mean ± SE, n = 3; ∗∗p < 0.01 vs. Ctrl). (D) Disappearance of the major sulfhydrated serum protein band after albumin depletion. Mouse serum was either left untouched or albumin-depleted with depletion reagent, and assayed for the level of protein sulfhydration using the dimedone switch method. EZ blue staining was performed to show the disappearance of the albumin band (arrowhead) without great influence on other protein bands. Note the obviously reduced sulfhydrated protein level at the albumin-depleted location (arrowhead). (E–F) Effect of the H_2_S donor NaHS on liver protein sulfhydration. Sulfhydration was assessed in liver lysates from mice administered with 15 mg/kg NaHS for 3 h (E) or after in vitro incubation with 100 μM NaHS for 1 h (F). Quantification of protein sulfhydration in the blot of E and F is shown at the right side (mean ± SE, n = 3; ∗∗p < 0.01 vs. Ctrl). (G–J) Alterations in serum and liver sulfhydration levels in doxorubicin (DOX) toxicity models, renal ischemia-reperfusion injury, and TNBS-induced colitis. Serum and liver lysates were collected from mice treated with 25 mg/kg DOX for 72 h (G, H), subjected to renal ischemia/reperfusion (I), or administered intrarectal TNBS for 24 h (J). Densitometric analysis of band intensities is presented to the right of the blots (mean ± SE, n = 3; ∗p < 0.05, ∗∗p < 0.01 vs. Ctrl). EZ blue staining of gels, confirming equal protein loading in each lane, is presented in Supplementary Fig. 4.

To confirm the presence of sulfhydrated proteins in serum, we analyzed protein sulfhydration in mouse serum. Fig. 1A shows the presence of sulfhydrated protein bands in serum proteins, particularly around molecular weight (MW) of 65 kDa, corresponding to the predicted MW of albumin. Further analysis revealed that the level of sulfhydrated serum proteins could be enhanced by intraperitoneal administration of NaHS, a widely used H_2_S donor (Fig. 1B). Consistently, direct incubation of serum with 100 μM NaHS caused an elevation in protein sulfhydration (Fig. 1C), suggesting that serum proteins are highly susceptible to sulfhydration. Collectively, these findings suggest that there exist sulfhydrated proteins in mouse serum, the level of which are upregulated in the presence of H_2_S.

Based on its molecular weight, abundance in serum, and high susceptibility to H_2_S modification, we hypothesized that albumin was the most intensively sulfhydrated protein in serum. To confirm this, we used a specific anti-albumin antibody and observed that the strongest band in EZ blue staining corresponded to albumin (Supplementary Fig. 1). Furthermore, depleting albumin from serum using an albumin depletion reagent resulted in the disappearance of the most prominent protein band in EZ blue staining, which was accompanied by a marked reduction in the signal of the most prominently sulfhydrated protein band (Fig. 1D). These findings indicate that albumin is the most prominently sulfhydrated protein in serum.

In addition to serum proteins, protein sulfhydrated (-SSH) groups were also detectable in the lysates of several organs, including the heart, colon, and liver (data not shown). Notably, the liver, which is known to be the primary source of H_2_S in vivo, exhibited a high basal level of sulfhydrated proteins, which could also be further upregulated upon exposure to H_2_S donor NaHS in vivo and in vitro (Fig. 1E and F).

Given that –SSH groups are highly reactive with reactive oxygen species (ROS), particularly under oxidative stress conditions [13,16], we investigated the levels of sulfhydrated serum proteins in several pathological conditions, including DOX-induced cardiotoxicity, renal ischemia-reperfusion injury, and TNBS colitis. These models were chosen because serum protein oxidation has been previously described in renal ischemia-reperfusion injury and TNBS colitis in our recent studies, while DOX-induced serum protein oxidation has been reported by others and confirmed in this study, as detailed later in the results [21,22,30]. As shown in Fig. 1G，I and J, serum sulfhydrated protein levels were significantly reduced in all three models. Additionally, in mice treated with DOX, a decrease in liver sulfhydrated protein levels was also observed (Fig. 1H). These findings demonstrate that sulfhydrated proteins are present in vivo and are regulated by H_2_S levels and oxidative status.

### 3.2 Preparation and characterization of sulfhydrated albumin (–SSH–Alb)

**Fig. 2:**
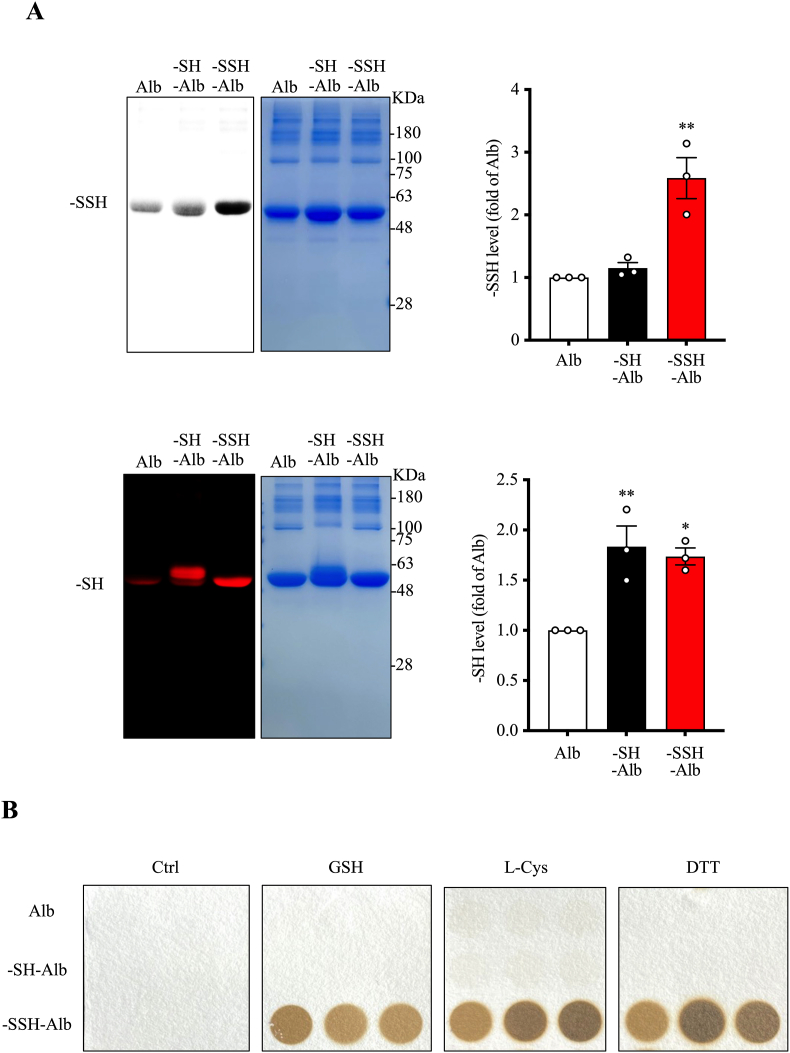
**Characterization of sulfhydrated albumin (-SSH-Alb).** (A) Quantifying sulfhydryl (-SH) and sulfhydrated (-SSH) groups in differentially modified albumin. The levels of –SH and –SSH groups in modified albumin preparations were determined and quantified. The signal intensities from the blots are shown to the right (mean ± SE, n = 3; ∗p < 0.05, ∗∗p < 0.01 vs. Alb). (B) H_2_S release from –SSH–Alb under reducing conditions. Differentially modified albumin (10 mg/ml) was incubated with 10 mM glutathione (GSH), 10 mM l-cysteine (L-Cys), or 5 mM dithiothreitol (DTT) for 2 h. Released H_2_S was detected using H_2_S test paper. Note the dark/brown circles indicating H_2_S production in the wells containing –SSH–Alb and reducing agents but not in the wells containing –SH–Alb.

To investigate the pathophysiology and pharmacological functions of sulfhydrated proteins, we prepared –SSH–Alb in vitro. The process involved reductive treatment with DTT to expose sulfhydryl (-SH) groups, oxidizing –SH with H_2_O_2,_ and subsequent treatment with H_2_S donor NaHS to generate –SSH–Alb [16,31]. The generated albumin was confirmed to contain –SSH groups by detecting sulfhydration with the dimedone switch method and H_2_S formation under reductive conditions. As illustrated in Fig. 2, the generated albumin possessed a high level of –SSH groups and produced H_2_S in the presence of reductive GSH, L-Cys, and DTT. These results thus indicate a successful induction of sulfhydration in albumin.

Because the procedure used to prepare sulfhydration in albumin was associated with the formation of –SH groups. We also prepared a –SH control by treating albumin with DTT to distinguish the effects of –SSH from –SH. As shown in Fig. 2A and B, DTT-treated albumin exhibited a higher level of free –SH groups than albumin control. Unlike –SSH–Alb, –SH–Alb could not produce H_2_S under reductive conditions.

It is worth mentioning that, to ensure that the observed effects from –SSH–Alb were not due to residual NaHS, we have throughly dialyzed the –SSH–Alb during the preparation process. Given that NaHS is a rapid H_2_S donor, releasing H_2_S almost instantaneously. Any residual NaHS or H_2_S would have been completely removed during the process of dialysis over a span of more than 3 days. Furthermore, we confirmed that the filtrate derived from the prepared –SSH–Alb did not release H_2_S (Supplementary Figs. 2A and B). Moreover, –SSH–Alb precipitated using trichloroacetic acid (TCA)/acetone retained the ability to release H_2_S (Supplementary Fig.2C). These observations thus excluded the potential influence of residual NaHS in the prepared –SSH–Alb.

### 3.3 -SSH-Alb retains, transmits, and amplifies H_2_S signaling

**Fig. 3:**
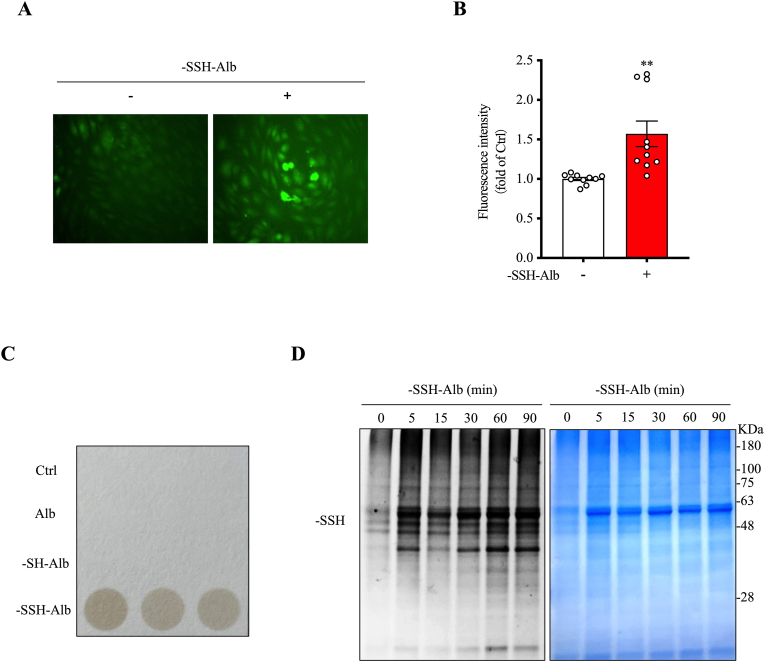
**-SSH-Alb increases intracellular H_2_S levels and induces protein sulfhydration.** (A, B) Effect of –SSH–Alb on intracellular H_2_S levels. Human umbilical vein endothelial cells (HUVECs) were treated with 2 mg/ml –SSH–Alb for 6 h, and intracellular H_2_S levels were visualized using a fluorescent probe Hsip-1DA probe (A). Fluorescence intensity was quantified and is presented in (B) (mean ± SE, n = 10 cells; ∗∗p < 0.01 vs. Ctrl). (C) -SSH-Alb-mediated H_2_S generation. HUVECs were incubated with 10 mg/ml –SSH–Alb or controls for 24 h, and H_2_S release was measured. (D) –SSH–Alb-induced protein sulfhydration. HUVECs were treated with 5 mg/ml –SSH–Alb for the indicated times, and cell lysates were analyzed for protein sulfhydration levels.

Using the prepared –SSH–Alb, we tested whether it mediated H_2_S signaling. For this purpose, cultured endothelial cells were used because of the direct contact with serum proteins in vivo. Fig. 3A and B show that adding –SSH–Alb to the culture significantly increased intracellular levels of H_2_S, as indicated by the markedly enhanced cellular fluorescence after adding a specific H_2_S probe. The H_2_S-releasing capacity assay and the level of cellular protein sulfhydration also confirmed the result. The addition of –SSH–Alb into cultured endothelial cells resulted in the formation of a brown-colored circle in H_2_S test paper (Fig. 3C), indicating the production of H_2_S, it also elevated the cellular level of protein sulfhydration (Fig. 3D). Interestingly, these changes were –SSH–Alb-specific and not observed in cells treated with the normal and –SH–Alb controls.

**Fig. 4:**
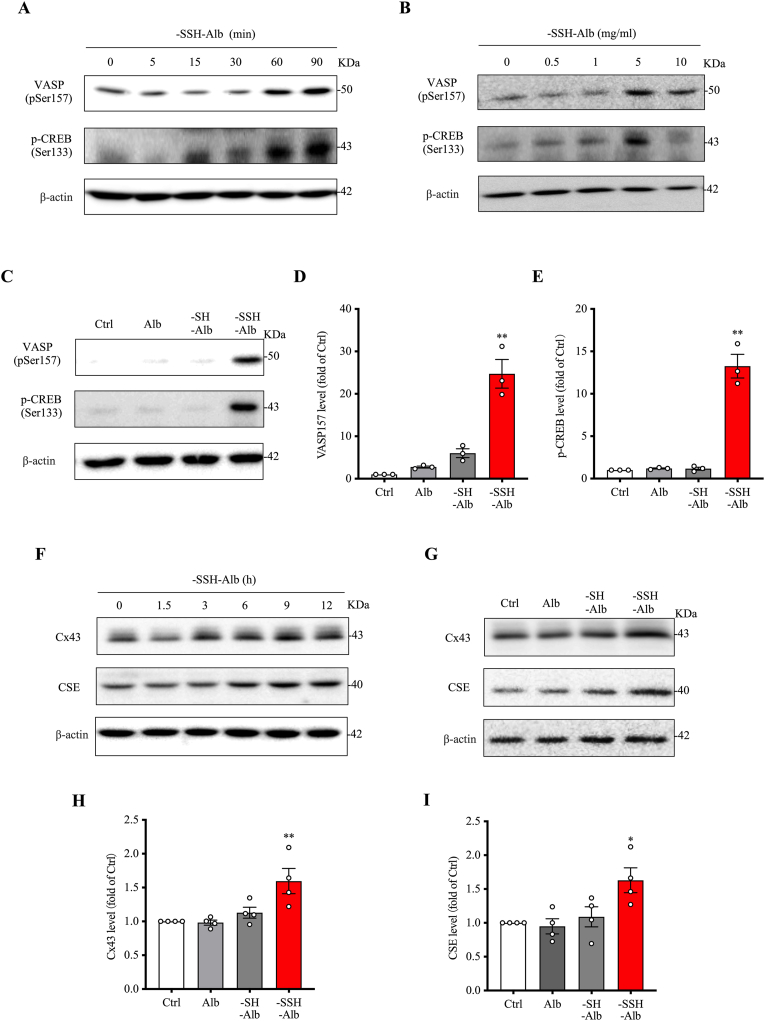
**-SSH-Alb activates the cAMP signaling pathway and stimulates Cx43 and CSE expression.** (A, B) Effect of –SSH–Alb on the cAMP signaling pathway. Cells were treated with 5 mg/ml –SSH–Alb for the indicated times (A) or with increasing concentrations of –SSH–Alb for 1 h (B). Cell lysates were analyzed by western blotting for the phosphorylation of VASP and CREB. (C–E) Effects of differentially modified albumin on the cAMP-PKA pathway. Cells were treated with 5 mg/ml of various albumin preparations for 1 h. VASP and CREB phosphorylation levels were assessed by western blotting (C), and the band intensities were quantified (D, E) (mean ± SE, n = 3; ∗∗p < 0.01 vs. Ctrl). (F) Effect of –SSH–Alb on Cx43 and CSE expression in HUVECs. Cells were treated with 1 mg/ml –SSH–Alb for the indicated times, and cell lysates were analyzed for Cx43 and CSE expression by western blotting. (G–I) Effect of differentially modified albumin on Cx43 and CSE expression. HUVECs were treated with 1 mg/ml of various albumin preparations for 9 h. Cx43 and CSE levels were assessed by western blotting (G), and the corresponding band intensities were quantified (H, I) (mean ± SE, n = 4; ∗p < 0.05, ∗∗p < 0.01 vs. Ctrl).

The H_2_S-releasing property of –SSH–Alb prompted us to test its influence on endothelial signaling. To this end, we examined the cAMP signaling pathway known to be activated by H_2_S and mediates many biological functions of H_2_S [27,32,33]. Fig. 4A and B show that –SSH–Alb induced VASP phosphorylation at Ser157, indicative of PKA activation. This effect was rapid, observable as early as 1 h after its addition, and time-/concentration-dependent. In further support of the activation of PKA, –SSH–Alb also induced phosphorylation of another PKA substrate CREB similarly (Fig. 4A and B). Furthermore, these actions were also specific to the –SSH group. It was observed only in –SSH–Alb, but not –SH– and control Alb (Fig. 4C–E).

To determine whether PKA activation could lead to changes in endothelial function, we examined the expression of Cx43, a PKA-controlled gene product that regulates many aspects of vascular functions [34,35]. As shown in Fig. 4F–I, –SSH–Alb elicited Cx43 expression in a –SSH–specific manner. Intriguingly, –SSH–Alb also stimulated the expression of CSE, the major H_2_S-synthesizing enzyme in endothelial cells. Collectively, these observations indicate that –SSH–Alb releases H_2_S, activates cAMP signaling, and elevates the H_2_S-synthesizing enzyme, suggesting that –SSH–Alb not only serves as a reservoir for H_2_S but also transmits and amplifies H_2_S signals.

### 3.4 -SSH-Alb suppresses inflammatory cell responses

**Fig. 5:**
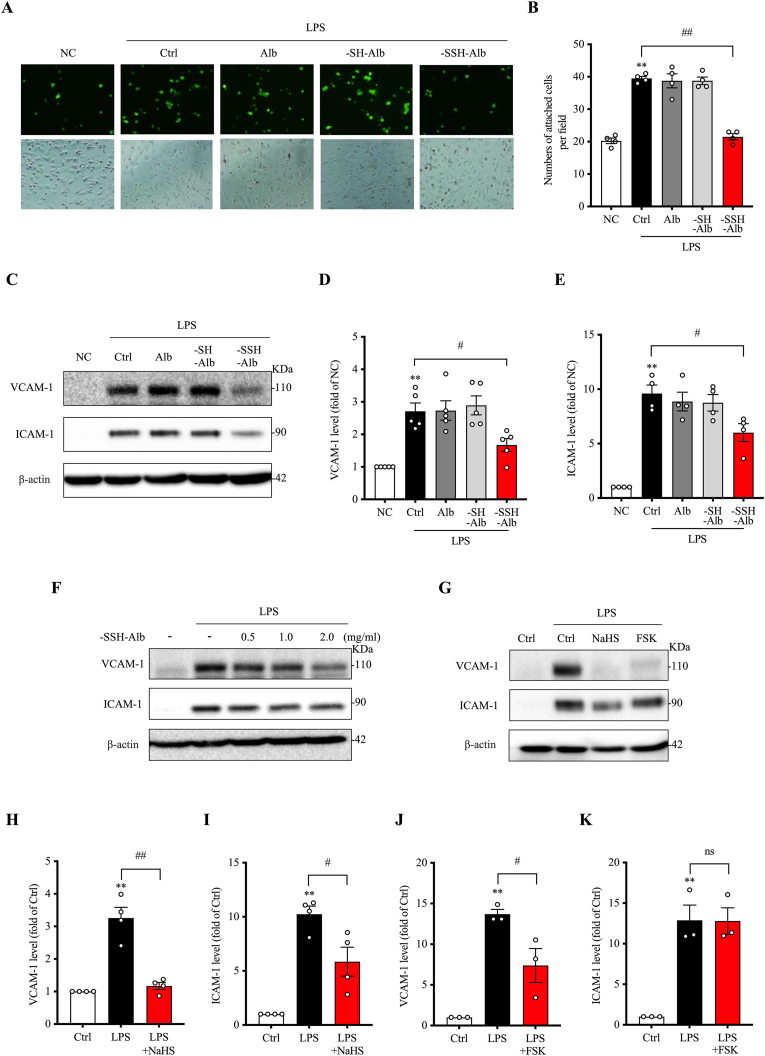
**-SSH-Alb inhibits macrophage adhesion to endothelial cells and suppresses LPS-induced adhesion molecule expression.** (A, B) Effects of differentially modified albumin on macrophage adhesion to endothelial cells. HUVECs were pretreated with 1 mg/ml of various albumin preparations or left untreated (NC, positive control) for 30 min, followed by stimulation with 500 ng/ml LPS for 6 h. Calcein-AM-labeled bone marrow-derived macrophages (BMDMs) were added and allowed to adhere for 30 min. Adherent macrophages were quantified by counting fluorescent cells (B) (mean ± SE, n = 4; ∗∗p < 0.01 vs. NC; ^##^p < 0.01). (C–F) Effects of differentially modified albumin or increasing concentrations of –SSH–Alb on endothelial adhesion molecule expression. HUVECs were pretreated with 1 mg/ml of various albumin preparations or increasing concentrations of –SSH–Alb for 30 min, followed by stimulation with 500 ng/ml LPS for 6 h. VCAM-1 and ICAM-1 expression in cell lysates were assessed by western blotting (C, F), and the band intensities of (C) were quantified (D, E) (mean ± SE, n = 4–5; ∗∗p < 0.01 vs. NC; ^#^p < 0.05). (G–K) Effects of NaHS and forskolin (FSK) on endothelial adhesion molecule expression. HUVECs were pretreated with 1 mM NaHS or 10 μM FSK for 30 min, followed by LPS stimulation for 6 h. VCAM-1 and ICAM-1 levels were assessed by western blotting (G), and the band intensities were quantified (H–K) (mean ± SE, n = 3–4; ∗∗p < 0.01 vs. Ctrl; ^#^p < 0.05, ^##^p < 0.01, ns: not significant).

We then tested whether, similar to H_2_S and other cysteine persulfide [1,5,36], –SSH–Alb could also have anti-inflammatory actions. For this purpose, we examined its effects on LPS-induced macrophage adhesion to endothelial cells, a key molecular event in the inflammatory process [37]. Fig. 5A and B show that LPS stimulation significantly enhanced macrophage adhesion to cultured endothelial cells, as demonstrated by the increased number of the Calcein-AM- prelabelled fluorescent macrophages. However, the increment was wholly abolished in the presence of –SSH–Alb.

Since macrophage-endothelial adhesion involves adhesion molecule expression and macrophage activation, we assessed the influence of –SSH–Alb on these molecular events. Fig. 5C–F shows that LPS induced the expression of adhesion molecules VCAM-1 and ICAM-1 in endothelial cells. The induction was significantly suppressed by –SSH–Alb. Further analysis revealed that the effects of –SSH–Alb on VCAM-1 expression were fully mimicked by the H_2_S donor NaHS and the cAMP activator forskolin. However, the effect of –SSH–Alb on ICAM-1 was only partially replicated (Fig. 5G–K), suggesting that an alternative mechanism other than the PKA pathway may also be involved.

**Fig. 6:**
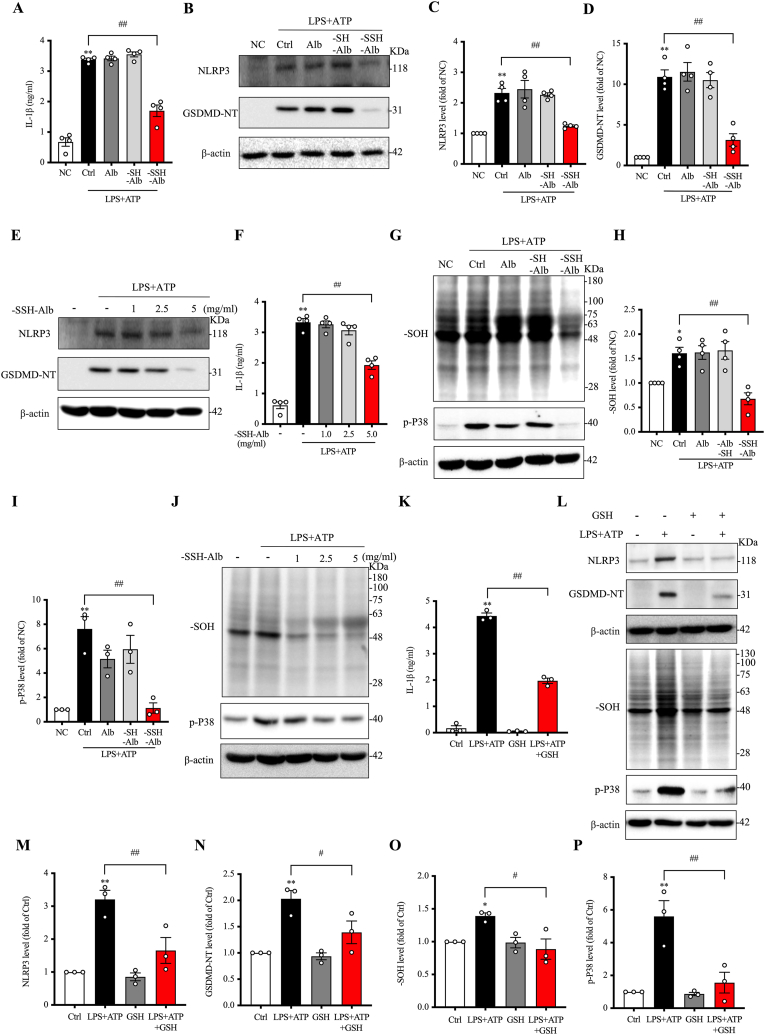
**-SSH-Alb inhibits inflammasome activation and pyroptosis in macrophages.** (A–F) Effects of differentially modified albumin or increasing concentrations of –SSH–Alb on LPS/ATP-induced inflammasome activation and pyroptosis. BMDMs were pretreated with 5 mg/ml of various albumin preparations or increasing concentrations of –SSH–Alb for 30 min, followed by stimulation with 10 μg/ml LPS for 5.5 h and 5 mM ATP for an additional 30 min. IL-1β release in the culture supernatant was measured by ELISA (A, F). Cellular lysates were analyzed for NLRP3 and GSDMD-NT by western blotting (B, E), and band intensities of (B) were quantified (C, D) (mean ± SE, n = 4; ∗∗p < 0.01 vs. NC; ^##^p < 0.01). (G–J) Effects of differentially modified albumin or increasing concentrations of –SSH–Alb on LPS/ATP-induced oxidative stress. Cells were treated as described above. Cellular lysates were analyzed for –SOH (sulfenic acid) modification and phosphorylated P38 (p-P38) by western blotting (G, J), and band intensities of (G) were quantified (H, I) (mean ± SE, n = 3–4; ∗p < 0.05, ∗∗p < 0.01 vs. NC; ^##^p < 0.01). (K–P) Effect of GSH on LPS/ATP-induced pyroptosis and oxidative stress. BMDMs were pretreated with 5 mM GSH or left untreated for 1 h, followed by stimulation with 10 μg/ml LPS for 5.5 h and 5 mM ATP for 30 min. Culture medium and cell lysates were collected and analyzed for IL-1β, NLRP3, GSDMD-NT, –SOH, and p-P38. Quantitative data are shown in (K, M − P) (mean ± SE, n = 3; ∗p < 0.05, ∗∗p < 0.01 vs. Ctrl; ^#^p < 0.05, ^##^p < 0.01).

We also investigated the impact of –SSH–Alb on macrophage activation. For this purpose, bone marrow-derived macrophages were cultured, and the influence of –SSH–Alb on LPS plus ATP-induced inflammasome activation was examined. Fig. 6A and F show that –SSH–Alb potently suppressed LPS/ATP-induced IL-1β production. Consistently, it inhibited inflammasome activation and macrophage pyroptosis, as revealed by the reduced levels of NLRP3 and GSDMD-NT (Fig. 6B–E). These effects were also concentration and –SSH group-dependent.

Given the reported antioxidative actions of –SSH groups and the central role of ROS in inflammasome activation [[38], [39], [40]], we examined the influence of –SSH–Alb on cellular redox status. Fig. 6G–J shows that stimulation of cells with LPS plus ATP elicited oxidative stress, as indicated by the increased sulfenic acid (-SOH) formation and p38 activation. In the presence of –SSH–Alb, these changes were completely abolished. In support of the critical involvement of oxidative stress in these inflammatory reactions, we also treated cells with thiol antioxidant 10.13039/501100022272GSH. We found that it also potently suppressed these inflammatory changes (Fig. 6K–P). These observations indicate that –SSH–Alb inhibits inflammatory responses and macrophage activation by modulating cellular redox status.

### 3.5 -SSH-Alb attenuates doxorubicin-induced cardiotoxicity and intestinal injury

**Fig. 7:**
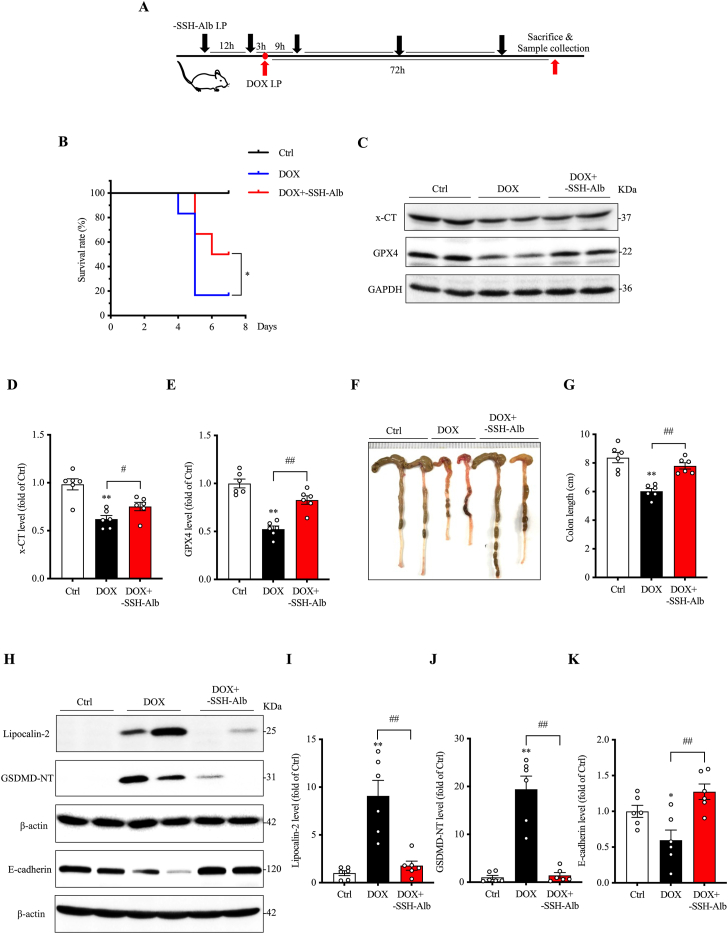
**-SSH-Alb alleviates doxorubicin (DOX)-induced cardiotoxicity and intestinal injury.** (A) Schematic diagram of the experimental design. For acute cardiotoxicity induction, mice received a single intraperitoneal injection of DOX (25 mg/kg) or normal saline (NS). –SSH–Alb (1.5 g/kg) was administered five times at 12–24 h intervals. Mice were euthanized 72 h after DOX treatment, and tissue samples were collected. (B) Effect of –SSH–Alb on DOX-induced mortality. Mice received a single intraperitoneal injection of DOX (25 mg/kg), and mortality was monitored for 7 days (mean ± SE, n = 6; ∗p < 0.05). (C–E) Effects of –SSH–Alb on DOX-induced cardiac ferroptosis. Heart tissue lysates were analyzed for ferroptosis markers (x-CT, GPX4) by western blotting (C), and band intensities were quantified (D, E) (mean ± SE, n = 6; ∗∗p < 0.01 vs. Ctrl; ^#^p < 0.05, ^##^p < 0.01). (F, G) Effects of –SSH–Alb on DOX-induced colon shortening. Colon was photographed (F) and measured for its length (F), and data are presented in (G) (mean ± SE, n = 6; ∗∗p < 0.01 vs. Ctrl; ^##^p < 0.01). (H–K) Effects of –SSH–Alb on DOX-induced colon injury and barrier dysfunction. Colon tissue lysates were analyzed for injury markers (lipocalin-2, GSDMD-NT) and the adherens junction protein E-cadherin by western blotting (H), and band intensities were quantified (I–K) (mean ± SE, n = 6; ∗p < 0.05, ∗∗p < 0.01 vs. Ctrl; ^##^p < 0.01).

Recent studies suggest that H_2_S deficiency contributes to doxorubicin (DOX)-induced cardiotoxicity [41]. Consistent with this, we observed reduced protein sulfhydration in serum and liver of DOX-treated mice (Fig. 1G and H)**.** These observations prompted us to investigate whether –SSH–Alb could mitigate DOX toxicity. Fig. 7A outlines the experimental design. Mice received –SSH–Alb before and after DOX administration. This treatment significantly reduced DOX-induced mortality by day 7. The mortality rate in the –SSH–Alb-treated group was 50 %, compared to 83 % in the DOX group (Fig. 7B). This reduction in mortality correlated with a significant attenuation of cellular damage, as indicated by markers of ferroptosis [42,43]. The decreased expression of x-CT and GPX4 observed in the DOX group was significantly reversed by –SSH–Alb treatment (Fig. 7C–E).

Given the reported role of intestinal injury in exacerbating DOX-induced cardiotoxicity and mortality [[44], [45], [46]], we also assessed the effects of –SSH–Alb on DOX-induced intestinal damage. –SSH–Alb significantly ameliorated DOX-induced macroscopic changes in the colon, reducing bleeding, edema, and shortened colon length (Fig. 7F and G). Furthermore, –SSH–Alb attenuated DOX-induced colonic injury, pyroptosis, and barrier dysfunction, as evidenced by decreased levels of the injury marker lipocalin-2 and the pyroptosis marker GSDMD-NT and increased levels of the adherens junction protein E-cadherin (Fig. 7H–K).

These findings demonstrate that –SSH–Alb protects against DOX-induced cardiotoxicity and intestinal injury.

### 3.6 -SSH-Alb improves local and systemic oxidative stress

**Fig. 8:**
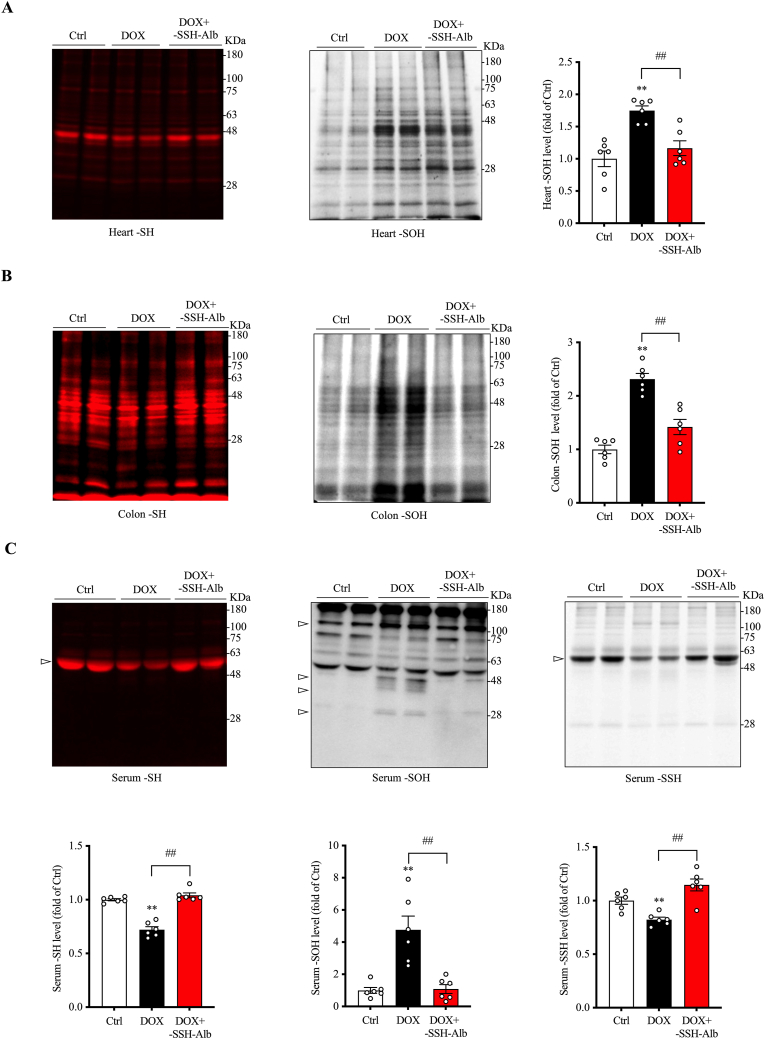
**-SSH-Alb treatment improves local and systemic oxidative stress.** (A, B) Effects of –SSH–Alb treatment on –SH (thiol) and –SOH (sulfenic acid) levels in heart and colon tissues. Heart and colon tissue lysates were analyzed for –SH and –SOH levels. Data are presented as mean ± SE (n = 6; ∗∗p < 0.01 vs. Ctrl; ^##^p < 0.01). (C) Effects of –SSH–Alb treatment on systemic oxidative status and serum sulfhydration levels. Serum levels of –SH, –SOH, and –SSH were measured. Quantitative results of the bands are presented below the blot and expressed as mean ± SE (n = 6; ∗∗p < 0.01 vs. Ctrl; ^##^p < 0.01). Note the difference in band intensity at the arrow-indicated location. The bands with MW between 28 and 48 were quantified for densitometric analysis of –SOH bands. EZ blue staining of gels, confirming equal protein loading in each lane, is presented in Supplementary Fig. 4.

Given the central role of oxidative stress in cell damage and inflammation, we assessed oxidative status in the heart, colon, and serum. Fig. 8A and B show that DOX administration significantly elevated protein –SOH formation in the heart and colon while decreasing protein –SH levels, indicating the induction of oxidative stress in the affected organs. –SSH–Alb treatment markedly attenuated these oxidative changes. Similarly, –SSH–Alb also prevented DOX-induced serum protein oxidation. It potently inhibited –SOH formation and –SH reduction in serum proteins. Notably, it also reversed the reduced levels of serum sulfhydration (Fig. 8C). In this case, the most pronounced changes in serum proteins were observed at the location of albumin.

These observations suggest that –SSH–Alb treatment ameliorates local and systemic oxidative stress and reverses sulfhydration deficiency under conditions of DOX toxicity.

## 4 Discussion

**Fig. 9:**
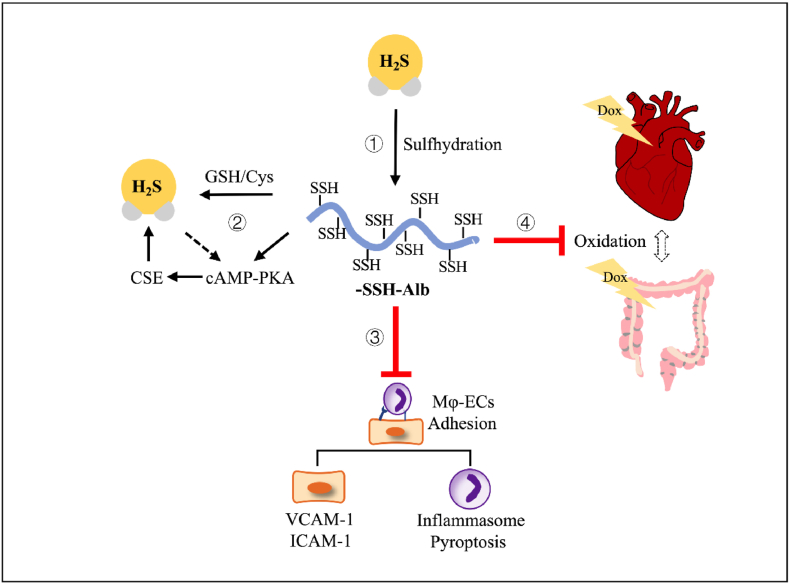
**Schematic depiction of the physiological and pharmacologic roles of -SSH-Alb.** –SSH–Alb, present in serum, is regulated by H_2_S levels and redox status. It acts as a reservoir, carrier, and amplifier of H_2_S signaling via releasing H_2_S, stimulating the cAMP pathway, and promoting CSE expression. It also possesses potent anti-inflammatory effects by inhibiting macrophage-endothelial cell interactions via suppressing adhesion molecule expression and attenuating inflammasome activation. In vivo, –SSH–Alb mitigates local and systemic oxidative stress, thereby protecting against DOX-induced cardiotoxicity and intestinal injury.

In this study, we demonstrated the presence of dynamically regulated sulfhydrated serum proteins in circulation, which transmitted and amplified H_2_S signaling. The sulfhydrated serum protein exhibited significant anti-inflammatory and antioxidative properties, which could be exploited to treat DOX-induced organ injury. Our findings reveal a novel mechanism of systemic H_2_S signaling mediated by circulating serum proteins, underscoring their important physiological and pharmacological potential. The summary of the findings of this study has been schematically depicted in Fig. 9.

Intracellular protein sulfhydration has been extensively studied and shown to mediate numerous biological and pharmacological functions of H_2_S. Our current research expands upon this understanding by revealing the dynamic regulation of sulfhydrated proteins in the extracellular space in response to both physiological and pharmacological H_2_S stimulation. Albumin is a key target for sulfhydration because of its abundance in serum, the readily available –SH group (-Cys34), and hepatic origin. Indeed, albumin was the main protein that was sulfhydrated in basal and H_2_S exposure conditions. Apart from the effect of H_2_S, albumin sulfhydration can also occur during its synthesis via a recently discovered CARS2-mediated enzymatic pathway [19,20,47]. This novel mechanism directly sulfhydrates newly synthesized proteins during post-translational modification, providing an alternative route for sulfhydrating albumin and other proteins. Beyond serum albumin, our findings revealed the widespread presence of sulfhydrated proteins in various organs, whose levels were dynamically regulated by both H_2_S concentration and redox status. In models of oxidative stress and H_2_S deficiency, protein sulfhydration levels were diminished, consistent with reports of decreased protein sulfhydration during aging [25]. These observations suggest a complex interplay between protein sulfhydration, H_2_S, and redox balance, with –SSH–Alb potentially serving as a valuable biomarker reflecting in vivo oxidative stress and H_2_S levels and having broader physiological implications.

A significant finding of our study was that –SSH–Alb functioned not only as a carrier of H_2_S but also as an integral component of H_2_S signaling through multiple mechanisms. As an H_2_S reservoir, it released the gaseous mediator in the presence of reducing agents or cellular components. Furthermore, it may facilitate long-distance H_2_S signal transmission, which differs from H_2_S and most available pharmacological donors, which have a limited diffusion range and only exert transient effects. Perhaps most importantly, –SSH–Alb amplified H_2_S signaling. It activated the cAMP-PKA pathway, which we and others have previously documented to be critically involved in the upregulation of H_2_S-synthesizing enzyme CSE [27,33]. As the pathway regulates a wide range of physiological functions [48] and mediates many classical H_2_S effects, such as vasodilation and inhibition of platelet activation [7], it is conceivable that this property of –SSH–Alb could have significant implications. The exact mechanisms underlying the actions of –SSH–Alb remains to be clarified. It could be related to the released H_2_S or –SSH group-mediated interaction with other important structural and functional molecules.

Our findings also demonstrated that –SSH–Alb exhibits a potent anti-inflammatory effect. It markedly suppressed macrophage-endothelial cell adhesion, adhesion molecule expression, and inflammasome activation. This effect of –SSH–Alb could be, at least in part, attributed to the released H_2_S and cAMP pathway activation, as it was partially mimicked by the H_2_S donor NaHS and the cAMP activator forskolin. Interestingly, –SSH–albumin appeared to exert a more potent inhibition on ICAM-1 than NaHS and forskolin. This outcome may suggest the potential involvement of additional mechanisms beyond H_2_S release and cAMP activation. More detailed analysis will be necessary in the future. It is worth mentioning that several studies have highlighted the critical role of sulfidation in regulating inflammation. A recent study by Salti et al. showed that macrophage activation was associated with a marked increase in cellular protein persulfidation [49], which, in turn, rendered macrophages more resistant to oxidative-inflammatory stress. This observation indicates an anti-inflammatory property of persulfidated cellular proteins. In line with this view, another study by Zhang et al. revealed that the cysteine-based small molecular polysulfide donor possesses potent anti-inflammatory effects in response to LPS-induced inflammatory responses and endotoxin shock [36]. This protective effect has been shown to be mediated via inhibiting Toll-like receptor 4 (TLR4) signaling. The results from our study suggest that, in addition to small molecular cellular polysulfides, H_2_S-modified extracellular serum proteins also exert potent anti-inflammatory actions, thus emphasizing the importance of extracellularly modified proteins in the body's defense against inflammation.

-SSH-Alb also exhibited a potent antioxidative effect in this study. –SSH–Alb significantly reduced intracellular protein oxidation and P38 activation in cultured BMDMs. In addition, in vivo administration of –SSH–Alb counteracted DOX-induced systemic oxidative stress. The effect of –SSH–Alb could be closely related it anti-inflammatory and cell-protective actions. It has been extensively reported that oxidative stress acts upstream of NF-κB activation and inflammasome assembly [40,50]. Antioxidants, such as GSH, used in this investigation could effectively prevent inflammatory cell responses. As -SSH groups have an even higher reactivity with free radicals compared to –SH groups, it may possess more potent antioxidant capacity than –SH–Alb [13,16]. Indeed, in this study, we observed that –SSH–Alb, but not –SH–Alb, activated the cAMP-signaling pathway and exerted a potent inhibition on oxidative stress and inflammation.

The anti-inflammatory and antioxidative properties of –SSH–Alb were further verified in the complex pathophysiology of DOX toxicity, where both oxidative stress and inflammation are critically involved. Moreover, an implication of H_2_S deficiency in DOX toxicity has also recently been reported [41]. Consistently, we also observed a reduced serum level of protein sulfhydration. –SSH–Alb treatment, however, improved DOX-induced mouse death, multiple organ damage, and systemic oxidative status. It prevented DOX-induced ferroptotic changes in the heart. The mechanism involved could be multiple, including improved systemic and local oxidative status, reduced local and systemic inflammatory situations, elevated cellular defense against ferroptotic cardiac injury, etc.

It is especially worth mentioning that the therapeutic actions of –SSH–Alb were associated with reduced cell injury in the gut. Given that recent studies have highlighted the importance of the gut-heart axis in DOX-induced mortality [44,46], the simultaneous protection of cardiac and intestinal damage could provide –SSH–Alb a unique advantage over other treatments.

Several studies have shown that H_2_S donors exhibited therapeutic effects on DOX cardiotoxicity [41,51,52]. The treatment with –SSH–Alb offers several distinct advantages, including extended H_2_S half-life and controlled H_2_S release, thus providing more therapeutic efficacy. The simultaneous antioxidant and anti-inflammatory properties of –SSH–Alb may help address multiple pathological processes concurrently. Furthermore, as albumin is a natural carrier protein with an established safety profile, it presents a promising platform for therapeutic development. The systemic distribution of albumin enables multi-organ protection, as evidenced by its beneficial effects on both cardiac and intestinal tissues in our DOX toxicity model.

Our findings could significantly impact our understanding of H_2_S biology and clinical applications. From a mechanistic perspective, our study provides novel mechanistic insight into systemic H_2_S signals. Protein sulfhydration in serum protein provided a way to store, transmit, and amplify H_2_S signals. From a diagnostic perspective, –SSH–Alb levels could be a good biomarker for H_2_S bioavailability and potentially indicate disease progression in H_2_S-deficient conditions. Therapeutically, our work suggests that –SSH–Alb could be developed as a pharmacological H_2_S donor, applied in various conditions characterized by H_2_S deficiency, including cardiovascular diseases, neurodegeneration, and aging-related disorders [25,53]. The protection against chemotherapy-induced toxicity, as shown in this study, suggested that it could be used to reduce adverse effects in cancer treatment. Of note, sulfhydration is a general phenomenon applicable to almost all cysteine-containing proteins. The biological effects of sulfhydrated albumin as shown in this study are likely also shared by other cysteine-containing proteins. In this context, our findings could have broader implications.

It is also worth mentioning that, in comparison to traditional H_2_S donors such as NaHS, –SSH–Alb may have several advantages, which makes it a promising candidate for therapeutic applications. First, –SSH–Alb offers the potential for sustained H_2_S release. This is due to its requirement for reductive conditions to liberate H_2_S and the structural localization of –SSH groups within less accessible regions of the protein. Second, as a native serum protein, albumin exhibits high biocompatibility and minimal toxicity. Third, –SSH–Alb uniquely combines the intrinsic biological properties of albumin with the therapeutic effects of H_2_S offering a multifunctional therapeutic agent capable of addressing multiple pathological processes simultaneously. Finally, unlike NaHS, –SSH–Alb does not generate toxic byproducts during H_2_S release, processing high safety and applicability in clinical settings. These attributes positions –SSH–Alb as a promising candidate for addressing conditions associated with oxidative stress, inflammation, and other H_2_S-regulated pathologies.

Our study also has limitations. While we have demonstrated the ability of –SSH–Alb to activate the cAMP pathway and suppress inflammatory responses, the precise fundamental molecular mechanisms underlying these effects require further elucidation. It is unclear whether mechanisms beyond H_2_S release contributed to the observed actions. Our preliminary studies indicated enhanced binding of –SSH–Alb to endothelial cells compared to unmodified albumin (Supplementary Fig. 3**)**, suggesting a potential direct interaction with cell surface molecules. Additionally, the –SSH group, known for its high reactivity, can participate in reactions with various biological molecules. For instance, persulfides, including –SSH–Alb, can transfer sulfane sulfur to other thiols, forming disulfides or react with sulfenic acids to generate protein-bound polysulfides [54].Furthermore, persulfides readily react with metal centers, such as those found in metalloproteins, potentially modulating their activity [55]. They can also scavenge reactive oxygen and nitrogen species, acting as antioxidants [56]. These diverse reactions of the –SSH group could have significant implications for cellular function. Future studies will be crucial to determine how these H_2_S-independent mechanisms contributed to the biological functions of –SSH–Alb.

In conclusion, our study establishes –SSH–Alb as a novel mediator of systemic H_2_S signaling and demonstrates its therapeutic potential through multiple mechanisms. This study opens new avenues for understanding extracellular H_2_S signaling and developing targeted therapies for H_2_S deficiency or oxidative stress conditions. The unique properties of –SSH–Alb, including its extended half-life, controlled H_2_S release, and multi-modal therapeutic effects, position it as a promising candidate for treating various pathological conditions. Future studies will reveal additional applications and mechanistic insights, potentially revolutionizing our approach to treating H_2_S-deficient and oxidative stress-related diseases.

## CRediT authorship contribution statement

**Yijun Xu:** Writing – original draft, Methodology, Investigation, Formal analysis, Data curation. **Yang Sui:** Methodology, Investigation. **Rui Jiang:** Methodology, Investigation. **Xin Wang:** Methodology. **Mika Suda:** Resources, Methodology, Funding acquisition. **Manabu Niimi:** Resources, Methodology. **Zhimin Mao:** Resources, Methodology. **Zhen Zhang:** Resources, Methodology. **Shao-Ling Zhang:** Resources, Funding acquisition. **Jianglin Fan:** Resources, Project administration, Funding acquisition. **Jian Yao:** Writing – review & editing, Validation, Supervision, Project administration, Funding acquisition, Conceptualization.

## Declaration of competing interest

The authors have declared that no conflict of interest exists.

## Data Availability

Data will be made available on request.
