# Supplementary material for: Sulfhydrated albumin transmits H2S signaling and ameliorates DOX-induced multiorgan injuries
Source: Redox Biol. 2025 Apr 8;83:103631. doi: 10.1016/j.redox.2025.103631 (PMC12018206; doi:10.1016/j.redox.2025.103631)
Supplement: Multimedia component 1 [file mmc1.pdf]

Supplementary Fig. 1

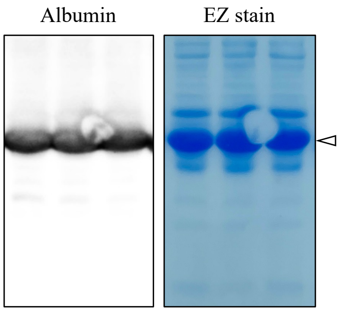

Fig. S1. Detection of serum albumin with an anti-albumin antibody. Denatured serum samples were separated by SDS-PAGE, transferred to PVDF membrane and immunoblotted for albumin with an anti-mouse albumin antibody. Note that albumin band in western blot (Left image) overlapped with the most predominant band in EZ blue staining (right image).

Supplementary Fig. 2

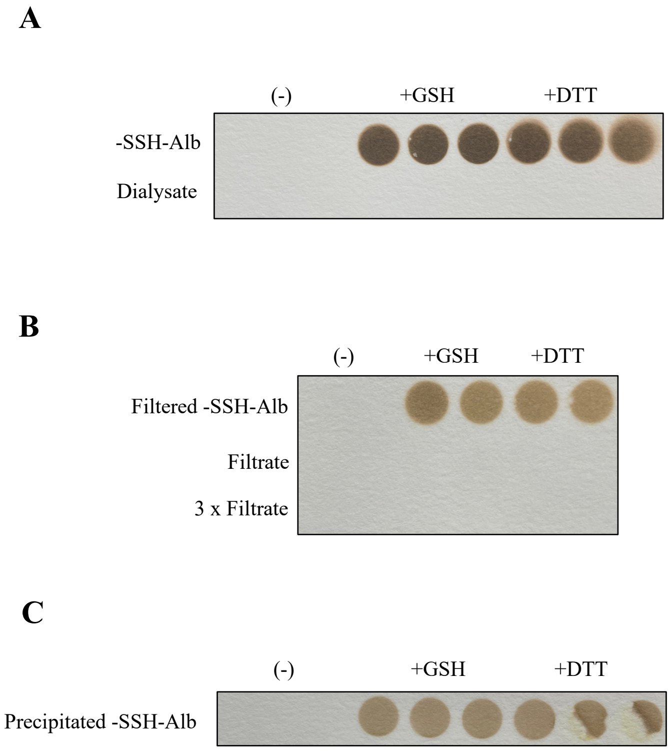

Fig. S2. Exclusion of the possible H_2S release from the residual NaHS in -SSH-Alb solution. (A) Detection of H_2S release from -SSH-Alb and final dialysate. A 10 mg/ml -SSH-Alb solution and an equal volume of final dialysate were added to a 96-well plate, and H_2S release was detected in the presence or absence of 5-10 mM GSH/DTT. Note no H_2S release from the dialysate. (B) Detection of H_2S release between -SSH-Alb and the filtered solution. The prepared -SSH-Alb solution was centrifuged in a ultrafiltration tube (10K MWCO) at 13,200 rpm for 20 min to separate -SSH-Alb and filtrate. The collected Alb and filtrate were assayed for their capacity to release H_2S under the same volume or with the volume of the filtered solution 3 folds over the volume of Alb (3xfiltrate). (C) H_2S release from precipitated -SSH-Alb. TCA/acetone precipitation was performed to remove all possible residual chemicals in -SSH-Alb. The precipitated protein was assayed for H_2S release. Note the H_2S release from precipitated -SSH-Alb under the reductive conditions. These results thus excluded the possible contamination of NaHS in the prepared -SSH-Alb solution.

Supplementary Fig. 3

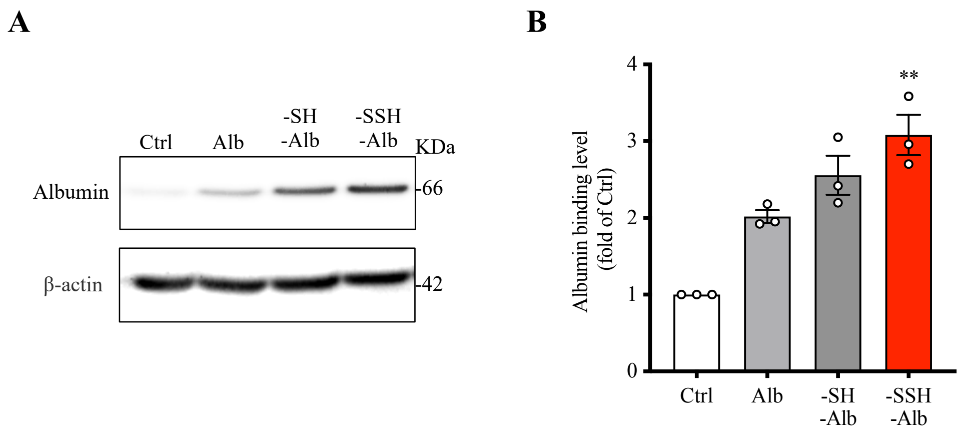

Fig. S3. Binding ability of differently modified albumin to cultured endothelial cells. Differently modified albumin at 2 mg/ml was added into cultured HUVECs and co-incubated for 3 h. The unbound albumin was washed out with culture medium and the remaining albumin was exacted with lysis buffer and subjected to Western blot analysis for its level (A). The equal loading of the protein in each lane was verified with an anti-β-actin antibody. The densitometric data of the blot in A is shown in B (mean ± SE, n = 3; $^{**}p < 0.01$ vs. Ctrl). Note the significantly increased -SSH-Alb binding to the cultured endothelial cells.

Supplementary Fig. 4

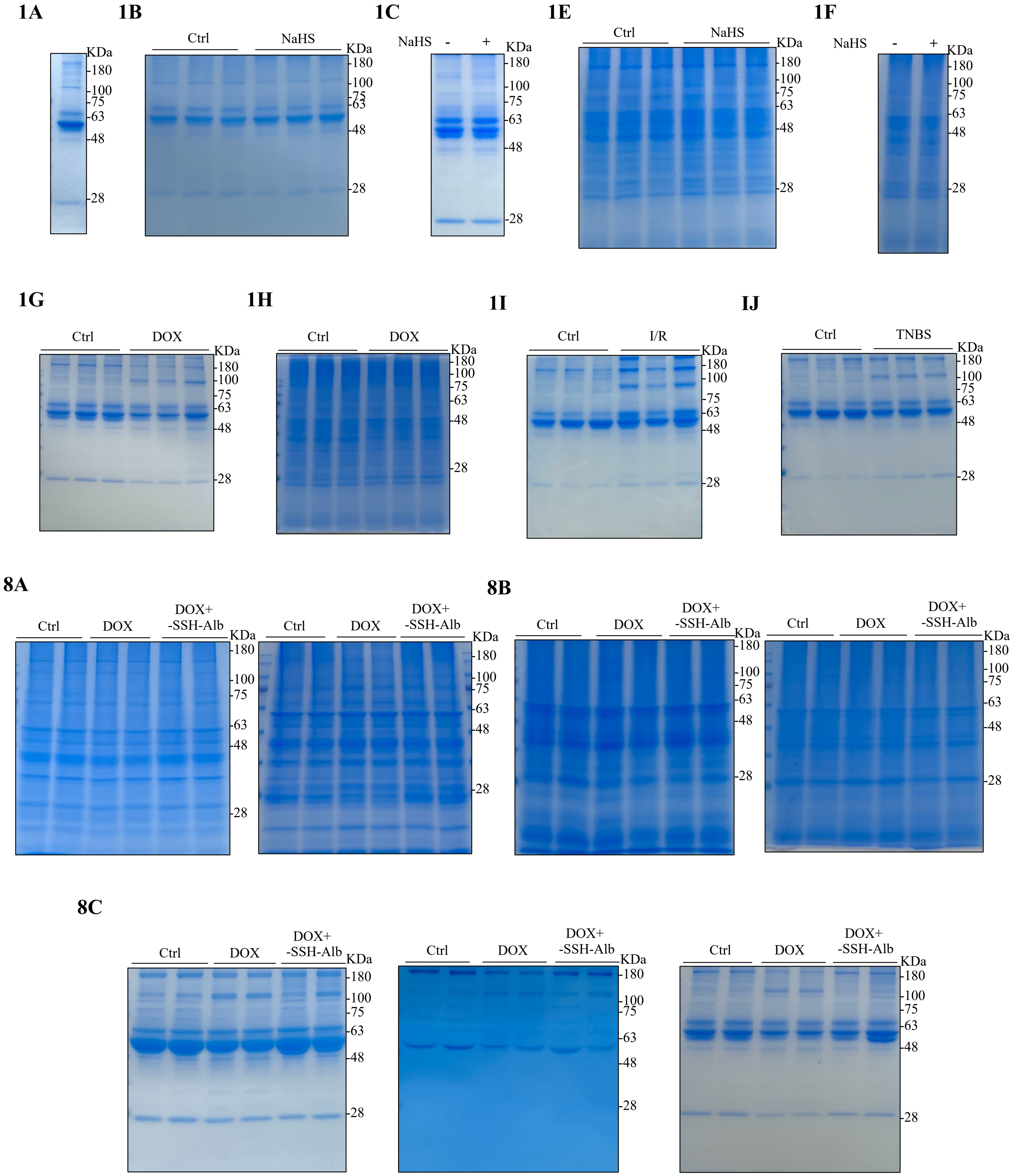

Fig. S4. The images of EZ blue staining of the gels presented in Figs. 1 and 8.
